# Smart Devices and Wearable Technologies to Detect and Monitor Mental Health Conditions and Stress: A Systematic Review

**DOI:** 10.3390/s21103461

**Published:** 2021-05-16

**Authors:** Blake Anthony Hickey, Taryn Chalmers, Phillip Newton, Chin-Teng Lin, David Sibbritt, Craig S. McLachlan, Roderick Clifton-Bligh, John Morley, Sara Lal

**Affiliations:** 1 Neuroscience Research Unit, School of Life Sciences, University of Technology Sydney, Broadway, Sydney, NSW 2007, Australia; blake.hickey1@my.nd.edu.au (B.A.H.); Taryn.Chalmers@uts.edu.au (T.C.); 2 School of Nursing and Midwifery, Western Sydney University, Penrith, NSW 2747, Australia; P.Newton@westernsydney.edu.au; 3 Australian AI Institute, University of Technology Sydney, Broadway, Sydney, NSW 2007, Australia; Chin-Teng.Lin@uts.edu.au; 4 School of Public Health, University of Technology Sydney, Broadway, Sydney, NSW 2007, Australia; David.Sibbritt@uts.edu.au; 5 Centre for Healthy Futures, Torrens University, Sydney, NSW 2009, Australia; craig.mclachlan@laureate.edu.au; 6 Kolling Institute for Medical Research, Royal North Shore Hospital, St Leonards, NSW 2064, Australia; roderick.cliftonbligh@sydney.edu.au; 7 School of Medicine, Western Sydney University, Penrith, NSW 2747, Australia; J.Morley@westernsydney.edu.au

**Keywords:** wearable devices, smart technology, electroencephalogram, heart rate variability, anxiety, depression

## Abstract

Recently, there has been an increase in the production of devices to monitor mental health and stress as means for expediting detection, and subsequent management of these conditions. The objective of this review is to identify and critically appraise the most recent smart devices and wearable technologies used to identify depression, anxiety, and stress, and the physiological process(es) linked to their detection. The MEDLINE, CINAHL, Cochrane Central, and PsycINFO databases were used to identify studies which utilised smart devices and wearable technologies to detect or monitor anxiety, depression, or stress. The included articles that assessed stress and anxiety unanimously used heart rate variability (HRV) parameters for detection of anxiety and stress, with the latter better detected by HRV and electroencephalogram (EGG) together. Electrodermal activity was used in recent studies, with high accuracy for stress detection; however, with questionable reliability. Depression was found to be largely detected using specific EEG signatures; however, devices detecting depression using EEG are not currently available on the market. This systematic review highlights that average heart rate used by many commercially available smart devices is not as accurate in the detection of stress and anxiety compared with heart rate variability, electrodermal activity, and possibly respiratory rate.

## 1. Introduction

Acute stress is a growing, unavoidable issue in contemporary society induced by physical and or emotional stressors, which physiologically can combine to trigger or exacerbate a wide variety of disease states [1,2]. In conjunction with negative emotions such as anxiety and depression, stress can increase cardiovascular disease risk, the leading cause of mortality worldwide [3]. Anxiety disorders are the leading mental health illness, with 264 million affected worldwide [4], with depression projected to be the second major cause of disability in the coming decade [5]. Further, the incidence of these mental health issues is increasingly developing in low- and middle-income countries [4]. Therefore, researchers are trying to create more compact, portable, and accurate technology to monitor stress and mental health status (depression or anxiety). Such devices will ultimately reduce morbidity and the economic burden on the health care system, as patients can seek help earlier or act to reduce symptoms or triggers [6,7].

Vital signs, neural activity (electroencephalogram ((EEG)), heart rate (electrocardiogram ((ECG)), skin temperature, and skin conductance response (electrodermal activity) can provide important information about an individual’s health status [8]. However, the challenge is how to make this information more readily available outside of the clinical environment using semi-validated, wearable devices that are tolerated by people and have regulatory approval for their stated purpose [6]. Within the past decade, the creation of commercially available smart devices and wearable technologies to monitor health has grown exponentially [9]. Many smart wearable devices are being developed, including smart textiles, pedometers, wearable EEG systems, smart watches with photoplethysmography, and many other devices that can non-invasively measure several health-related factors [8].

However, stress and mental illness are in a different paradigm and often difficult to monitor objectively, with the recent focus on the feasibility of creating technology capable of detection of mental states. Further, research over the last ten years has demonstrated that people are reluctant and find it uncomfortable to wear invasive or large intrusive devices for measuring health status [1,6]. Since smartphones and wearable devices are often carried on one’s person as an integral part of life in modern society, they are often chosen as the instruments to detect and monitor stress, anxiety, and depressive symptoms. This review focuses on both bulky wearables and sensor wearables; devices which are tolerable to the wearer, portable, and proposed to be capable of detecting stress, anxiety, and depression. What is currently known about wearable devices which measure these mental states is the primary focus of this review.

Specifically, this systematic review aims to answer the following questions:

1. What types of smart devices and wearable technologies are being used to detect or monitor depression, anxiety, and stress?
2. What physiological or other process(es) do smart devices and wearable technologies utilise to detect depression, anxiety, and stress?
3. Which of these devices have been manufactured and are available on the market as technology?

## 2. Materials and Methods

### 2.1. Selection Criteria for the Present Review

Review Period. This systematic review was limited to the articles identified using the search strategy outlined below to review wearable smart technologies’ most current developments to detect and/or monitor depression, anxiety, and stress, and how this is detected physiologically using such technology. The search was conducted initially on 27 December 2020 and updated on 4 January 2021, with a further updated search on 11 April 2021.

Types of studies and study design. Various study designs were included due to the low yield of studies. In addition, studies that only used machine learning without their use in a current wearable device or application were excluded.

Categories of effect. All studies were included in this review based on the primary search measures stated in the Primary Effects below. Studies were not excluded based on the reported method of investigation or research field.

Primary effects. The identification of wearable technologies and smart applications that detect or monitor fluctuations in mental health (specifically depression, anxiety, and stress) and the physiological measures used to detect these changes. The selection criteria for inclusion of articles were agreed upon by the collaborative expertise of the authors (B.H., T.C., P.N. and S.L.).

### 2.2. Literature Search Methods

Peer-reviewed journal publications with complete full text, regardless of study design and the country where the study was conducted, were the only articles included in this review. Year of publication was not included in the exclusion criteria due to the focus on modern technology, with the earliest included paper about this topic published in 2010. Studies published in a language other than English, or those structured as editorials, news releases, research highlights, or letters, review articles, commentaries, and technical papers, were excluded.

### 2.3. Electronic Database Search

The CINAHL, MEDLINE, Cochrane Central, and PsycINFO databases were searched. The primary search terms were limited to the title and abstract fields and included wearable device, wearable technology, smart device, and wireless device. Secondary search terms were limited to the title and abstract fields and included depression, anxiety, stress, and fatigue. The search structure for the Medline (Table 1), CINAHL Plus (Table 2), Cochrane Central (Table 3), and PsycINFO (Table 4) databases are provided below.

Selection of studies. Upon completion of the systematic database search, duplicate articles were discarded. Two authors (B.H. and T.C.) screened the remaining articles separately with use of Covidence systematic review software [10] which allows blind review of articles by each reviewer to prevent bias, with unanimity on the articles removed. No additional articles were added from external sources. Following inspection of titles and abstracts, articles were extracted using the criteria above. The remaining articles were subsequently sourced in full-text and critically appraised using the Joanna Briggs Institute Critical Appraisal Checklist [11], a 10-question checklist appraising the research methodology and level of bias of research articles. 

## 3. Results

**Figure 1:**
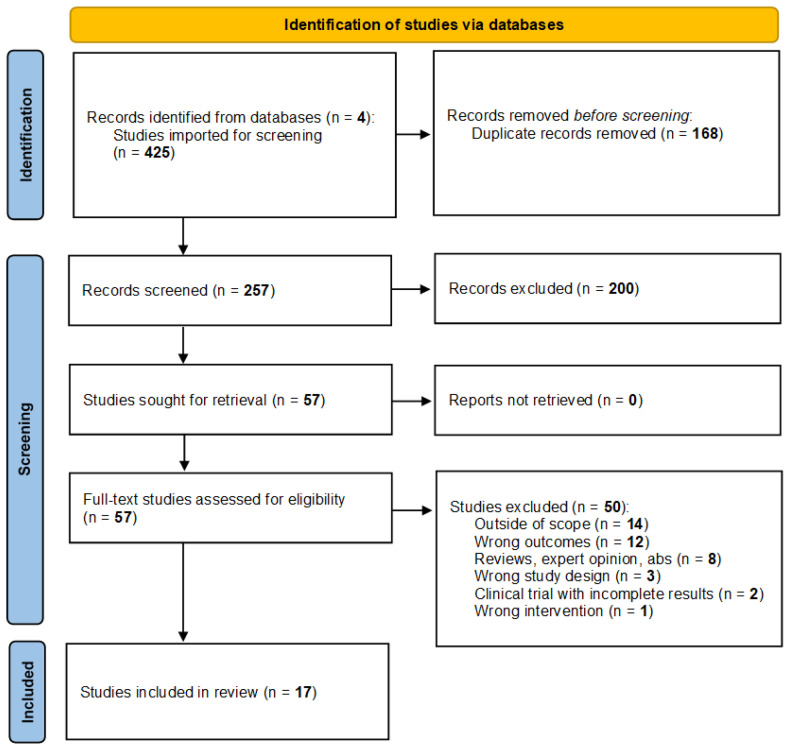
Flow diagram of the systematic search strategy and results conducted using Covidence systematic review software [10].

The overall systematic search strategy was conducted using Covidence systematic review software, which allows for collation of citations, records articles not suitable for review and the reasoning for rejection, and allows blind screening of articles between reviewers. The results of the systematic search process performed on Covidence are presented in Figure 1. A total of 567 studies were yielded by the systematic search across the four databases mentioned above, with no additional studies added outside of this search strategy. Of the identified studies, 223 duplicates were removed, leaving 342 studies. A further 265 studies were excluded following a review of their title and abstract, with studies excluded if the abstract did not mention the use of wearable technologies or smart devices for detection of stress, anxiety, or depression. When the search parameters anxiety, depression, and stress were used in addition to the primary search terms, the combined number of articles yielded was small, with considerable overlap of studies between search terms and databases. The search term “anxiety” yielded 91 articles for potential review; of these, most of the articles were either not experimental studies, did not use the wearable device to measure any physiological parameters, or anxiety was never assessed in the study. The search term “stress” yielded 121 journal articles, with the potential to be included in this review; of these, the majority were removed for not using the wearable device to measure physiological data relating to stress, with remaining papers removed for being review papers, articles with results not currently available, or articles outlining the methodology of studies yet to be completed. The search term “depression” yielded 97 journal articles, with the potential to be included in this review; of these, the majority were removed for not assessing depression in the experimental study or for not collecting physiological data which was used to assess for depressive symptoms.

Following a review of the 75 full-text studies remaining, 54 studies were excluded, primarily due to not having stress, anxiety, or depression as a measured outcome, lack of experimental results, the article focused primarily on the machine-learning aspect of a device, or the study used a paediatric population. The remaining 21 studies were critically appraised using the Joanna Briggs Institute Critical Appraisal Checklist and included in the review.

The key findings of these papers, including the type of wearable device, the physiological process used to detect anxiety, depression and/or stress, and whether the device is commercially available, are discussed below. A summary of the 21 included articles is presented in Table 5.

Of the 21 studies reviewed, five wearables measured HRV to detect stress, two wearables measured HRV and EEG concurrently to detect stress and anxiety, and two wearables measured fluctuations in HR to detect stress. Utilising the physiology of the integumentary system, three wearables measured either galvanic skin response (GSR) or electrodermal activity (EDA) to detect stress, and one device measured skin conductance and HR to detect stress. Depression was detected with two wearables which measured EEG signals, one wearable which measured HR and podometry, one wearable which measured activity using a podometer, and one wearable which recorded brain oxy-hemodynamic responses using functional near-infrared spectroscopy (fNIRS). A multiparametric garment was one wearable capable of measuring GSR, skin temperature, ECG, and respiratory rate to detect stress in soldiers. Finally, two very recent wearable devices measured respiratory rate to detect stress, but with one study reporting too few results.

## 4. Discussion

### 4.1. Anxiety

Anxiety is a common mental health issue, particularly in Australia where the prevalence is increasing [31]. It is defined as an unpleasant, emotional response out of proportion to a particular stressor (or even in the absence of), the response of which may or may not be prolonged, resulting in tension and physiological manifestations [32,33]. Episodes of anxiety are triggered from unnecessary stimulation of the hypothalamic–pituitary–adrenal axis, which stimulates the sympathetic limb of the autonomic nervous system (while simultaneously dampening the parasympathetic limb), which results in both psychological and physiological manifestations [32]. Of the latter, alterations in heart rate, respiratory rate and electrodermal activity reflect the function of the sympathetic nervous system [34]. Further, heart rate variability, which is calculated from the R–R interval, has been previously shown to represent the autonomic nervous system activity and is a good marker for stress and anxiety, with anxiety resulting in decreased R–R interval time and increased heart rate due to bolstered sympathetic response and reduced vagal inputs [35,36]. In a recent review by Elgendi and Menon (2019), the validity of using ECG parameters using wearable devices to detect different clinical diagnoses of anxiety was assessed [4]. The overall findings of experimental papers were conflicting and controversial, and the authors concluded that it was challenging to determine the impact ECG features had on determining anxiety with a need for more robust studies moving forward [4]. These cardiovascular measures, as well as respiratory and skin-related measurements, have been incorporated into wearable technologies that were assessed in the studies below.

### 4.2. Findings of This Review

Four studies [13,14,24,27] were identified for inclusion in this review based on the secondary search term “anxiety”. The study by Balconi et al. (2019) used wearable EEG and ECG (for subsequent HRV computation) devices, including either the Muse™ headband (InteraXon Inc., Toronto, ON, Canada) or the Lowdown Focus glasses (SmithOptics Inc., Clearfield, UT, USA) to determine the effects of mindfulness exercises on both an individual’s objective and subjective levels of stress and anxiety [14]. The study provided minimal detail on which EEG signatures were used on subjects, though the authors suggested that the wearable brain-sensing device has potential for promoting objective stress response by increasing awareness of EEG signatures of dysfunctional hyperactivation [14]. With respect to cardiovascular changes, HRV measures were reduced both at rest and during the stressor task, in conjunction with subjective decrease in stress and anxiety. Reduction of high-frequency components of HRV were found to be useful autonomic measures of the impact of stressors or stress-inducing situations and therefore have implications for the assessment of anxiety and stress [14,37]. Further, reduction in the high-frequency component of HRV (which is a marker of parasympathetic influence on cardiac activity) is consistent with the neurovisceral integration model of stress response [38], which outlines the physiological association between parasympathetic vagal activity and improved executive function (alluding to sympathetic function induced by stress and anxiety, dampening executive function).

Cardiac activity has been the predominant objectively measurable physiological parameter of anxiety in the literature; however, respiratory patterns have been reported to robustly indicate cognitive emotional stress [39,40]. The second article included in the present review, Smith et al. (2020), attempted to measure respiratory rate and variability to compare physiological parameters with subjective scores of anxiety and stress, using the only wearable device (Spire Stone (Spire Health, Stanford, California)) available at the time that could measure these parameters unobtrusively [27]. Despite the capability of the device in measuring respiratory rate and variability, there was a lack of compliance by subjects in the experimental group (they only wore the device 52% of the study days), despite the majority of subjects having reported high tolerability for the device. It was noted that breathing exercises are often implemented to regulate anxiety in people and were not assessed in the biofeedback model of the study [27], which may be worth investigating in the future, as slow, deep breathing is useful in reducing anxiety. The third article included in this review for anxiety, Alberts et al. (2020), also used the Spire Stone (Spire Health, Stanford, CA, USA)) and an adapted version, the Spire Health Tag respiratory monitor (Spire Health, San Francisco, CA, USA). Unlike the study by Smith et al. (2020), the Spire Stone was found to be tolerable in 90.3% of participants, with respiratory rate patterns found to be useful in detection of anxiety and stress [13]. Further studies testing respiratory rate and variability using wearable technology alongside subjective stress and anxiety results are required.

The relationship between the sympathetic nervous system and the integumentary is well-known, with this physiological relationship being used to detect anxiety, stress, and even depression. The fourth study included in the review for “anxiety”, Sano et al. (2018) using two sensors, compared the accuracy of skin conductance (SC), skin temperature (ST), and the three-axis acceleration using the wearable Q-sensor (Affectiva, Boston, MA, USA) and the Motion Logger (AMI, Ardsley, NY, USA) which records activity levels, in detecting mental health conditions and stress, with ST and SC being more useful in detection of stress and mental health conditions [24]. This is unsurprising, as SC has been considered a biomarker for stress [41] and also reflects the level of autonomic arousal, which can provide a stress index during wakefulness. With an accuracy of 87% and 78.3% for detecting poor mental health and depression, respectively, the dual sensor device Q-sensor (Affectiva) was a success in the study by Sano et al. (2018), as one of the first wearable devices to detect stress in a 24/7 daily life setting. Unfortunately, the findings of the three-axis acceleration, which can be used to estimate activity levels and sleep or wake patterns, was not the overall focus of this study [24], though many smart phones currently use this technology, which is useful in detecting depressive symptoms.

### 4.3. Stress

The bidirectional relationship between emotion and stress is well-known, with many papers reporting the influence emotion has over the autonomic nervous system (Kreibig et al., 2010). The physiological response from acute stress is often protective; however, chronic stress is known to facilitate numerous physical and mental health illnesses, which has a significant economic impact [17,28]. The understanding of chronic stress impact on the body has driven researchers to continue to develop new ways to detect and monitor stress, typically relying on the sympathetic nervous system physiological responses induced by stress, including changes in heart rate, heart rate variability, skin temperature, and conductance (van Kraaij et al., 2020). Algorithms developed based on these well-researched parameters have high accuracy for detecting stress more than 90% of the time in experimental conditions [42,43]. The use of various wearable devices and sensorised garments have been trialled to assess if they can accurately record the physiological responses created by sympathetic nervous system activity, using non-invasive cardiac, respiratory rate, skin conductance, and temperature [2,44].

According to a recent review, heart rate variability is the most studied [6] of all physiological parameters. This study provides a very succinct review of over 60 different wearable technologies, which assess a multitude of different physiological parameters including those mentioned in this review, with the addition of sleep and cognitive function [6]. This review article also contemporaneously reviews which wearable devices have been formally validated for use in research for stress (10%), with only 5% of the wearable technologies listed in the review having been formally validated as capable of accurately detecting health parameters [6].

Electrodermal activity has also gained favour as a marker of sympathetic nervous activity due to its emerging relationship neurophysiologically [45]. Skin conductance responses are associated with the ventromedial prefrontal cortex involved in anticipatory EDA responses, and the amygdala involved in EDA responds to the learned association between stimuli and reinforcement [45], with EDA now seen as an index of attention and not merely a measure of sympathetic activity.

### 4.4. Findings of This Review

A total of 15 studies were identified for inclusion in this review based on the search term “stress”. Similar to anxiety, the use of cardiac metrics, namely heart rate and heart rate variability, were the predominant physiological markers of stress detection in 10 of the 15 studies which detected stress [9,12,14,17,18,20,22,23,26,28]. It has been reported that altered HRV measurements are related to ANS dysregulation associated with many cardiovascular diseases including cardiac ischemia, myocardial infarction and heart failure, diabetes, and obesity, as well as mental health conditions including anxiety and depression [9,46]. Hernando et al. (2019), with use of the Apple Watch, reported that HRV measurements (in particular R–R interval series) are superior in detecting stress compared to HR alone, with most commercially available devices using average HR, which is heavily controlled by the autonomic nervous system and can also be drastically altered in certain physiological and pathological circumstances [9]. Further, in these situations where there is altered autonomic function (depression), this will be reflected in HRV metrics but not HR alone [47].

In the study by Rodrigues et al. (2020), the Vital Jacket^®^ (1-Lead, Biodevices S.A, Matosinhos, Portugal) was used to assess specific HRV metrics, namely the average of normal-to-normal intervals (AVNN), standard deviation of all normal-to-normal intervals (SDNN), root mean square of differences between successive rhythm-to-rhythm intervals (RMSSD), and low frequency/high frequency (LF/HF) ratio [23]. During stress, AVNN, RMSSD, and the percentage of successive R–R intervals that differ by more than 20 ms (pNN20) decreased, reflecting a depressed HRV, which is the expected response to stress [48]. Additionally, during stress a significant increase in the LF/HF ratio was reported, highlighting the impact of stress on the sympathovagal system [23]. These metrics were useful in identifying stressful situations, and promote the need for the production of quantified occupational health (qOHealth) devices to detect stress, as this study also reported that during stressful episodes, cognitive performance declines.

In the study by Huang et al., the Polar V800 Heart Rate Monitor (Polar Electro OY, Kempele, Finland), which monitors heart rate variability, was validated against ECG HRV under differing stressors, with high correlations. This study found that this wearable device is capable of monitoring stress to the same extent as an ECG, and therefore capable of detecting acute stress [18].

During acute stress, the limbic system and thalamus are activated by the cerebral cortex through the reticular activating system, which subsequently activates the hypothalamus, triggering an autonomic nervous system and endocrine response, resulting in catecholamine and cortisol secretion [49]. In the study by Hong et al. (2010), epinephrine, the stress response hormone, was unsurprisingly reported as having the highest correlation with qualitative stress levels [17]. Moreover, HRV index and LF/HF ratio were surprisingly more accurate in stress detection than cortisol [17], alluding that HRV metrics detected using wearable devices may be superior and more convenient than hormone and neurotransmitter analysis in detecting stress. HRV parameters are reported to be the most reliable in detecting stress, though many devices still use average HR alone, as reported below.

In studies that only examined HR [20,22] it was found that there was a significant difference between genders, with females having significantly higher average HR than males when exposed to occupational stress, when measured using an Apple Watch (Series 1, Apple Inc., Cupertino, CA, USA) [20]. Interestingly, Lucas et al. (2019) also commented that baseline cardiovascular fitness, determined by survey, had no significant impact on HR, which is the inverse of what is physiologically expected [20]. Further, in the study by Pakhomov et al. (2020), which used the Fitbit^®^ (no model specifics provided) to detect HR at baseline and during exposure to stressors, it was found that the Fitbit^®^ is capable of detecting stressors, with HR increasing an average of nine beats per minute [22]. Whilst the findings by Lucas et al. (2019) and Pakhomov et al. (2020) suggest HR may be useful in detecting stress, both studies were limited by the young age of their subjects; thus, the impact of comorbidities on HR, and therefore stress detection, may not be reflective of the general population [20,22]. The study by van Kraajj et al. (2020) supports this, using two separate wearables: an unspecified chest patch for HR measurement and a wristband (Chillband) for detecting activity, with the study reporting that there was a significant relationship between HR and the three-way interaction between chronic stress, gender, and circadian rhythm [28]. Further, it is known that maximum HR decreases linearly with age [50], with sleep and stress levels fluctuating majorly throughout life, which further supports this relationship. The influence of female gender over HR may require wearable devices to have HR scaled to accommodate for this physiological difference, though it appears that HRV metrics may make average HR detection obsolete.

EEG, as an adjunct to HRV in stress detection, was also assessed in two studies [12,14]. Asymmetric analysis of the frequency-band powers in the EEG, measured at the prefrontal cortex, has been previously used to detect stress [51]. The creation of a novel EEG and ECG system capable of simultaneously recording HRV features showed that EEG was more accurate (87.5%) in the detection of stress compared with EEG (77.9%) and HRV (75%) alone, thereby confirming that the simultaneous measurement of the EEG and HRV is more effective for stress detection when combined [12]. Whilst EEG is reported as more sensitive for stress detection in this study, its ability to be incorporated into a compact and visually appealing wearable device is still limited; however, the Muse™ headband is capable of doing this, though its tolerability as a wearable device is not known.

The physiology of the integumentary system in responding to stress is a well-understood phenomenon, with two studies [16,19] incorporating this physiology into a wearable device. Engelniederhammer et al. (2019), who used a sensor smart wristband (Bodymonitor™, Gesis Leibniz-Institute for the Social Sciences, Mannheim, Germany), reported that the EDA is the most simplistic and accurate indicator of emotional arousal, notably stress or aggression [16]. EDA is useful in the detection of stress but may pose challenges with respect to reliability of results in populations who have comorbidities such as diabetes mellitus or hyperthyroidism (though this can be overcome using models as outlined by Kim et al., 2020 [19]). The study by Kim et al. (2020) used a wearable Empatica wristband (E4, Empatica Inc., Boston, MA, USA) which recorded galvanic skin response (GSR) to detect stress in drivers, with an accuracy of 85.3% [19]. Further, the study reported that GSR sensors are currently the preferred method for stress detection, due to ease of setup, its compact nature, and overall simplicity when compared with EGG and ECG [19]. One study performed by Silva et al. (2020), using the Microsoft Smartband 2™, measured HRV in conjunction with skin conductance, which, when incorporated into a machine learning algorithm, could detect stress [26]. Multiple HRV parameters were significantly different during stressful conditions than baseline, notably mean R–R and PSS_13_ scores.

Another study by Seoane et al. (2014) suggested that multiparametric testing (including GSR, temperature, respiratory rate, and ECG) via a prototype wearable garment had superior accuracy in detection of stress than EDA. This formed part of the “Assessment in Real Time of the Stress in Combatants” project [25], which created a wearable garment capable of detecting physical and mental stress within military combat soldiers by monitoring HR, respiratory rate, and EDA [25]. Whilst the wearable device is capable of detecting stress, there was a high rate of error across the metrics, with almost twice as many GSR and skin temperature errors compared with ECG and respiratory rate [25]. This wearable device has prompted other researchers [2] to develop future wearable devices capable of detecting multiple stress-related metrics. In the study by Cho et al. (2017), the research team wanted to create wearable technology that measured photoplethysmograms, electrodermal activity, and skin temperature, with the aim of combining these parameters to accurately detect stress throughout the day [2]. Further, incorporating wearables such as this with feedback solutions to lower stress may aid in reducing the burden stress has on people in everyday life.

In one systematic review, it was found that electrodermal activity is useful in measuring neurocognitive stress, as skin conductance increases when individuals are stressed [52], which reported a wearable not identified with the above search terms. The “shimmer sensor” is a monitoring wearable sensor which uses EDA for stress monitoring, using two finger sensors, capable in one reported study of detecting stress in 86% of subjects; however, HRV and EEG data were also used in detection [52]. An additional study was also found outside of the search criteria which measured EDA to determine the level of pre-surgery stress, using the wrist wearable ADI-VSM (Analog Devices), with an accuracy of 85% [53].

A notable issue with EDA as a means of detecting stress was reported by Anusha et al. (2019), who reported that devices reliant on EDA data are prone to motion artifacts; further, varying pressure exerted on EDA electrodes related to the variable tightness of the wearable and movement of the hand and wrists may also distort the data in a major way, leading to potentially false readings [53].

### 4.5. Depression

The search term “depression” identified five studies [5,15,21,29,30] which assessed only depression, and one study [24] which assessed “mental health” broadly. EEG is a non-obtrusive, electrophysiological measure of the spontaneous electrical activity in the brain and is widely used to study antidepressant treatment responses due to its availability and low cost [54]. Two studies [5,15] reported the use of EEG in detection or monitoring of depressive symptoms. Cao et al. (2018) tested the response of depressive symptoms to ketamine by analysing EEG changes measured using a wearable forehead EEG device [15], the Mindo-4S Jellyfish (Eee Holter Technology Co. Zhubei District, Hsinchu, Taiwan). The theta and low alpha activity signatures were used as the EEG metrics in this study, which were significantly improved from baseline after ketamine treatment. In terms of neurophysiology, it has been reported that depressive disorders are correlated with a reduction in dorsolateral prefrontal cortex grey matter volumes, as well as unique directional changes in the prefrontal cortex [55]. Li et al. (2015) also used a single-electrode EEG (no specifics provided) to detect depressive symptoms, using specific classifiers, including k-nearest-neighbour (kNN), naïve Bayes (NB), logistic regression (LR), support vector machine (SVM), and random forest (RF) [5]. The kNN performed best out of the outlined classifiers, detecting mild depressive symptoms in 99.1% with the study concluding that a combination of linear and nonlinear EEG features proved to be effective in improving the accuracy of detecting depression; however, the sample size of this study was rather small [5]. One notable advancement in the wearable Mindo-4S Jellyfish by Cao et al. (2018) is the use of dry electrodes and the reduction in preparation time; due to the ease of wearing the device, this may eventuate into a means of monitoring depressive symptoms daily [15].

One study by Zhu et al. (2020) used a 16-channel wearable continuous-wave functional near-infrared spectroscopy (fNIRS) device model 1000 (United States) to measure brain oxy-hemodynamic (HbO) response. The accuracy of the fNIRS in accurate classification of depression was found to be 92.6% [30]. Further, this study also identified mean HbO, full width half maximum, and kurtosis as specific neuro-markers for predicting major depressive disorder across particular brain regions, notably the dorsolateral and ventrolateral prefrontal cortex [30]. The information provided by fNIRS and EEG devices is constantly improving the understanding of depression from a physiological stance, with further investigations of fNIRS in a larger sample size required.

The remaining two studies [17,29] looked at activity levels for the detection of depression using a wearable actigraph watch or smart watch with or without a smartphone. In the study by Zanella-Calzada et al. (2019), real-time measurements of behaviour, feelings, and activity were recorded using an Ecological Momentary Assessment [56], through use of smart phones and an actigraph watch; specifically, the Actiwatch (Cambridge Neurotechnology Ltd., Cambridge, UK, model AW4) [29]. This assessment is necessary for depression monitoring, as most depressive symptom monitoring methods rely on patient reports, which are commonly biased. When blindly selecting depressed subjects from non-depressed subjects, this method accurately detected depressed patients 86.7% of the time [29]. Inversely, it also detected non-depressed subjects in 91.9% of cases. Detecting depression based on the level of physical activity throughout a day through a smartphone may expedite new diagnoses or recurrences in people with depression.

In another study assessing physical activity, Narziev et al. (2020) selected five depression symptom factors which were extracted from the DSM-5 questionnaire, with mood, physical activity, sleep, social activity, and food intake (to ascertain appetite information) and monitored to detect depression using the developed “Short-Term Depression Detector” (STDD) framework, which used smart watch (Galaxy S3) sensors and Android smartphone [21]. Mood was determined by a combination of the above factors using machine learning. For the focus of this review, it was noted that the smart watch used a heart rate monitor and accelerometer to assess physical activity level in subjects, which is typically lower in depression. The study reported that the STDD framework and passive data collection had a strong correlation with the self-reported depression score, with the STDD having an accuracy of 96% in depressive group classification (Narziev et al., 2020). This study highlights the difficulties of objectively recognising depressive symptoms using wearable technologies and promotes the idea of using smartphone apps to gather metrics and qualitative data to assist in detecting depression.

## 5. Conclusions

The incidence of anxiety, depression, and chronic stress are increasing globally, as is the production of smart devices to help individuals monitor components of their health. Based off the wearable devices yielded by the aforementioned search terms, HRV was identified as the most useful physiological metric for detection of stress and anxiety [9,17,23]. Of devices on the market currently, most of them utilise average HR, which can monitor stress, albeit not as accurately as HRV parameters. Adjunctive EEG increases the accuracy of stress detection [12]; however, future studies need to assess the applicability of dual devices for long-term monitoring of chronic stress. EDA was considered a useful metric for detecting stress, reported by one author to be the preferred wearable due to simplicity and setup [19]; however, another author expressed some unreliability in results from EDA measurement through wearable devices due to motion artefact [52]. Detection of depression using wearable devices is an ongoing challenge, with wearable EEG [5,15] and accelerometers [21,29] currently used for detection, with the prior capable of detecting depression alone, whilst physical activity in a machine learning model can accurately detect depression. Further research into combining an accelerometer and EEG into one wearable device may yield promising results for depression diagnosis, though currently smartphone incorporation is heavily present within the literature for detection using subjective questionnaires and self-reporting [57].

Further research is needed into remote measurement technology, which can objectively measure disrupted sleep, reduced sociability, physical activity, changes in mood, prosody, and cognitive function—useful indicators of depression [58]. Passive remote measurement technology uses sensors in activity monitors and smartphones to gather data automatically, to assess if the wearer is demonstrating communication and activity patterns congruent with a depressive episode [59]. At the time of this review, this technology had no observable experimental data but, in the future, may be one of the leading technologies to detect depression. Wearable technologies will continue to evolve in their ability to provide the user with fast, health-related information. Whilst many of the aforementioned technologies are not advertised as “medical devices”, emerging devices such as smart watches do provide the wearer with a perception of their own health status, potentially influencing an individual’s attitude and response to their perceived health status; therefore, further investigation into validating wearable technologies and recording parameters on smart devices for interpretation would be highly beneficial.

A potential limitation of this study is the omitted devices not yielded using the search terms provided above, which subsequently may impede on the impact of conclusions made by this study. Technologies that were not included in this review may have been missed as a result of other authors’ choices of terminology, not consistent with our selected search terms. Further research into the incorporation of other devices, which are able to detect mental health changes and stress, not currently termed “wearables” may add to the conclusions drawn in this review.

With mental illness incidence continually climbing, the desire for technology capable of earlier detection or symptom management is highly desirable. While there has been a large amount of work conducted on wearable devices used in stress detection, further investigation into anxiety and depression detection is crucial in helping reduce the global impact of mental health issues, with multiparametric devices likely to provide the greatest likelihood for detection.

## Figures and Tables

**Table 1:** The search structure for the MEDLINE database.

Search Term	No. of Results
Step 1. Primary search term 1: Wearable device.ab,ti.	1496
Step 2. Primary search term 2: Wearable technology.ab,ti.	892
Step 3. Primary search term 3: Smart device.ab,ti.	1265
Step 4. Primary search term 4: Wireless device.ab,ti.	1429
Step 5. Combined primary search terms: Search term 1 or 2 or 3 or 4	2987
Step 6. Using only the secondary search terms below: - Depression.ab,ti - Anxiety.ab,ti - Stress.ab,ti	73,999 50,356 162,184
Step 7. Combined Step 5 and Step 6 (where Step 6 was): - Depression.ab,ti - Anxiety.ab,ti - Stress.ab,ti	34 24 113

**Table 2:** The search structure for the CINAHL Plus database.

Search Term	No. of Results
Step 1. Primary search term 1: Wearable device.ab,ti.	454
Step 2. Primary search term 2: Wearable technology.ab,ti.	463
Step 3. Primary search term 3: Smart device.ab,ti.	345
Step 4. Primary search term 4: Wireless device.ab,ti.	346
Step 5. Combined primary search terms: Search term 1 or 2 or 3 or 4	1358
Step 6. Using only the secondary search terms below: - Depression.ab,ti - Anxiety.ab,ti - Stress.ab,ti	35,468 25,877 42,329
Step 7. Combined Step 5 and Step 6 (where Step 6 was): - Depression.ab,ti - Anxiety.ab,ti - Stress.ab,ti	18 17 45

**Table 3:** The search structure for the Cochrane Central database.

Search Term	No. of Results
Step 1. Primary search term 1: Wearable device.ab,ti.	535
Step 2. Primary search term 2: Wearable technology.ab,ti.	300
Step 3. Primary search term 3: Smart device.ab,ti.	434
Step 4. Primary search term 4: Wireless device.ab,ti.	313
Step 5. Combined primary search terms: Search term 1 or 2 or 3 or 4	1394
Step 6. Using only the secondary search terms below: - Depression.ab,ti - Anxiety.ab,ti - Stress.ab,ti	78,922 53,821 60,314
Step 7. Combined Step 5 and Step 6 (where Step 6 was): - Depression.ab,ti - Anxiety.ab,ti - Stress.ab,ti	93 93 85

**Table 4:** The search structure for the PsycINFO database.

Search Term	No. of Results
Step 1. Primary search term 1: Wearable device.ab,ti.	164
Step 2. Primary search term 2: Wearable technology.ab,ti.	121
Step 3. Primary search term 3: Smart device.ab,ti.	123
Step 4. Primary search term 4: Wireless device.ab,ti.	79
Step 5. Combined primary search terms: Search term 1 or 2 or 3	435
Step 6. Using only the secondary search terms below: - Depression.ab,ti - Anxiety.ab,ti - Stress.ab,ti	76,926 62,904 72,329
Step 7. Combined Step 5 and Step 6 (where Step 6 was): - Depression.ab,ti - Anxiety.ab,ti - Stress.ab,ti	14 13 18

**Table 5:** Summary table of included research articles which utilise wearable technologies to detect stress, anxiety, and depression.

Study	Wearable Technology	Population	Objective	Primary Results
Ahn et al., 2019 [12]	Novel, wearable EEG and ECG head band that hangs behind both ears.	14 male subjects, young (mean age: 29.4 ± 3.3), healthy, with no reported comorbidities.	To develop a wearable device consisting of four sensors, with the capability of detecting stress using dual EEG and ECG.	Combined EEG and HRV were most accurate (87.5%) in detecting stress in healthy subjects, compared with EEG alone (77.9%) and HRV (75.0%).
Alberts et al., 2020 [13]	Spire Stone (Spire Health, San Francisco, CA, USA) and Spire Health Tag respiratory monitor (Spire Health, San Francisco, CA, USA) which monitor respiratory rate.	65 subjects (30 male, 35 female), middle-aged (mean age: 44.1 ± 8.7), in adults with chronic pain.	To assess the feasibility, acceptability, and efficacy of wearable respiratory monitoring for chronic pain and associated stress, depression, and anxiety.	The device was acceptable to 90.3% of subjects. The use of wearable respiratory monitoring may be useful in monitoring anxiety and stress.
Balconi et al., 2019 [14]	Wearable brain-sensing device, either the Muse™ headband (InteraXon Inc.) or the Lowdown Focus glasses (SmithOptics Inc.).	55 subjects (38 male, 17 female), young (mean age: 23.2 ± 1.8), with no known diagnosed psychiatric illness or cognitive impairment.	To use wearable devices in conjunction with mindfulness techniques to reduce overall stress and anxiety in a healthy population.	The use of wearable technology and mindfulness resulted in reduced perceived stress and anxiety. Further, HRV measures were reduced by the technology-mediated mindfulness exercise.
Cao et al., 2018 [15]	Wearable forehead EEG device the “Mindo-4S Jellyfish” (Eee Holter Technology Co.).	55 subjects (10 male, 45 female), middle-aged (mean age: ~mean age: 48), with treatment-resistant depression (TRD).	To determine the response to ketamine in patients with TRD using a wearable forehead EEG and Hamilton depression rating score.	Post-ketamine treatment EEG signatures in the prefrontal cortex showed improvements in EEG depressive signatures when compared with baseline EEG (*p* < 0.05).
Engelniederhammer et al., 2019 [16]	Sensor smart wristband (Bodymonitor) which monitors electrodermal activity (EDA) and skin temperature.	30 subjects (10 male, 20 female), young (mean age: 24.8 ± 0.7) with no known diagnosed psychiatric illness.	Using a wearable device to detect if invasion of individual personal space in crowded environments elicits an emotional response, including stress and aggression.	Aversive emotional responses (i.e., stress, anger, fear) detected by EDA-based emotion response type, were increased when personal space was encroached upon on busy streets.
Hernando et al., 2018 [9]	Apple Watch compared against the Polar H7 chest device (Polar Electro Oy) both of which detect HRV.	20 subjects, young in age with no reported comorbidities (little demographic information provided).	To validate the Apple Watch in terms of HRV derived from the R–R interval series against the R–R interval provided by the Polar H7 chest device and validated in both a stressed and relaxed state.	HRV indices from the R–R interval series reflected changes brought on by mild stress compared with relaxed states. HRV from the Apple Watch was not significantly different to the Polar H7 chest device.
Hong et al., 2010 [17]	Dual electrode ECG device the Biopatch, worn on the precordium.	29 (25 male, 4 female) healthy, young (mean age = 29.62 ± 5.28) participants, no known cardiovascular or neurological disease.	To verify the reliability of a wearable ECG device to detect stress, by correlating HRV to qualitative stress indices, cortisol, and catecholamine levels.	HRV parameters had high concordance correlations with stress. Catecholamines had the highest correlation with qualitative stress level. HRV had weak correlation with cortisol.
Huang et al., 2021 [18]	Heart rate variability measured using Polar V800 Heart Rate Monitor (Polar Electro OY, Kempele, Finland),	40 (17 male, 23 female) elderly (aged 65–79 years old) participants, with no established cognitive impairment or neurological disease.	To investigate the validity of the Polar V800 heart rate monitor for assessing R–R intervals and to evaluate differences in HRV when under physical or cognitive stressors.	HRV parameters on the wearable device were highly correlated with ECG HRV, and were capable of adequately measuring HRV under different stressors.
Kim et al., 2020 [19]	Galvanic skin response (GSR) was recorded using Empatica wristband (E4, Empatica Inc.).	9 subjects (no further demographics provided).	To develop a statistical model that accurately classifies driving stress by monitoring GSR.	Classification accuracy of the device to detect stress during driving was 85.3%.
Li et al., 2015 [5]	Single electrode EEG (no specifics provided).	36 subjects (24 male, 12 female), 9 of which had mild depression.	To create a device which accurately and objectively detects depression and related risk factors.	99.1% detection of mild depression using k-nearest neighbour kNN classifiers on EEG.
Lucas et al., 2019 [20]	Apple Watch (series 1) to track HR changes.	23 subjects (16 male, mean age 30.1, 7 female, mean age 30.6), majority not physically active, with no chronic comorbidities.	To monitor changes in HR while exposed to occupational stress and to determine if gender or physical activity influences this change in HR.	Baseline HR significantly increased during a shift when compared to baseline, with females’ HR significantly higher than males (*p* < 0.001). Physical activity level had no significant impact.
Narziev et al., 2020 [21]	Android smartphone and smartwatch (Gear S3 Frontier) accelerometer, significant motion, and step count data, to assess physical activity.	20 subjects (no demographic data) with either no, mild, moderate, or severe depression (PHQ-9).	To develop a Short-Term Depression Detector (STDD) framework that utilises a smartphone and smartwatch to continuously monitor self-reported symptoms and passive sensing data, respectively. Mood was ascertained by a combination of physical activity measures and mean HR.	Using a machine learning model, subjects’ passive sensing data (mood, physical activity, and sleep) was used to classify depression category and had high correlation to the self-reported depression score. The STDD had an accuracy of 96 ± 2.8% in depressive group classification.
Pakhomov et al., 2020 [22]	Fitbit^®^ (equipped with photoplethysmography sensor).	18 subjects (4 male, 14 female), young (mean age: 20.6 ± 2.0) that were non-smokers, not pregnant and with no comorbidities effecting HR.	To evaluate if wearable technology can detect physiological responses to stress when an individual is exposed to both stress-inducing scenarios and stress in everyday life.	Baseline HR was significantly lower than the HR recorded during a stressor (as identified by EMA surveys), up to ~9 beats higher. Concluded that Fitbit may be useful in identifying stressors in everyday life.
Rodrigues et al., 2018 [23]	Vital Jacket^®^ (1-Lead, Biodevices S.A), a medical-grade wearable ECG device and actigraph.	11 subjects (8 male, 3 female), mean age 46.7 ± 5.9, with no known cardiovascular diagnoses.	To assess the impact that stress has on cognitive performance in air traffic controllers and if this decreased performance was related to an autonomic stress response.	The TSST stress task resulted in significant changes in several HRV parameters and subjective stress level (STAI-six item) compared to baseline readings, as well as a decline in cognitive performance.
Sano et al., 2018 [24]	Two sensors on each wrist: Q-sensor (Affectiva) to measure skin conductance (SC), skin temperature (ST), three-axis acceleration (ACC), and Motion Logger to measure acceleration and ambient light data.	201 college students (129 male, 72 female), young (aged 18–25) collected across 3 years (2013–2015).	To determine how accurately physiological and behavioural measures recorded off wearable devices could detect stress and poor mental health (anxiety/depressive symptoms), and to evaluate which of these physiological or behavioural measures were most accurate.	Wearable sensor features (SC, ST) were more accurate in classifying poor mental health (87%) and stress (78.3%) than mobile phone and modifiable behavioural modalities when using machine learning.
Seoane et al., 2014 [25]	Multiparametric sensorised garment, containing one: GSR, Temperature unit, ECG, Thoracic Impedance recording unit, Sensorised glove, upper-arm strap, chest strap system, and six textrodes and smart phone for voice recording.	42 subjects (no demographic information provided).	The Assessment in Real Time of the Stress in Combatants (ATREC) project aimed to create a comprehensive wearable system to detect the real-time emotional, physical, and mental stress load of soldiers during military combat by monitoring various physiological parameters.	HR and respiratory rate, collected via ECG and electrical bioimpedance measurements from the thorax, respectively, were more useful in the assessment of stress than GSR, skin temperature, or speech.
Silva et al., 2020 [26]	Microsoft Smartband 2™, which measures skin conductance, body temperature, heart rate variability, calorie intake and expenditure, sleep patterns, and quality.	82 Portuguese (63 male, 19 female) young (17–38 years old) medical students.	To assess the level of stress experienced by medical students during examination by monitoring of HRV parameters.	Multiple HRV parameters were significantly different during stressful condition than baseline.
Smith et al., 2020 [27]	Spire Stone (Spire Health) which tracks respiratory rate and variability. It identifies stress, which is relayed to a smartphone.	169 subjects (76 male, 93 female), young (mean age: 33.2 ± 7.8).	To determine if mental health outcomes can be improved via a wearable stress management intervention and breathing biofeedback.	Of those that partook in the stress management intervention, 28.2% felt a reduction in stress and anxiety. The respiratory data was unavailable due to poor subject compliance in wearing the device.
vanKraaij et al., 2020 [28]	Chillband (wristband) to measure skin conductance and temperature, and ePATCH™ Extended Holter monitor.	328 subjects (186 male, 142 female), mean age 38.9 ±10.2.	This study is part of the Stress in Work Environment (SWEET), with the aim to determine if chronic stress influences HR over time and to assess if gender or age modulates this effect.	There is a significant relationship between HR and the three-way interaction of chronic stress, gender, and the circadian harmonic. Female gender was found to be associated with higher heart rates.
Zanella-Calzada et al., 2019 [29]	Actiwatch (Cambridge Neurotechnology Ltd.) detects activity levels.	5895 subjects from the “Depresjon Dataset” were used. No demographic data was given.	To use feature extraction of motor activity level data to detect depressed subjects accurately.	Subjects with depression were accurately detected in 86.7% of cases and those without depression were identified in 91.9% of cases.
Zhu et al., 2020 [30]	Brain oxy-hemodynamic (HbO) responses were recorded using a 16-channel wearable continuous-wave functional near-infrared spectroscopy (fNIRS) device model 1000, (United States).	31 subjects, 14 with clinically diagnosed major depressive disorders (6 male, 8 female), and 17 healthy controls (6 male and 11 female), aged 20 to 80 years.	This study aimed to assess the feasibility of fNIRS to assess and classify depression using a motor rehabilitation task.	Subjects with depression were accurately classified in 92.6% of subjects. This study also identified mean HbO, full width half maximum and kurtosis, as specific neuromarkers, for predicting major depressive disorders across dorsolateral and ventrolateral prefrontal cortex.

ECG = electrocardiogram; EEG = electroencephalogram; EMA = ecological momentary assessment; HR = heart rate; HRV = heart rate variability; SC = skin conductance; ST = skin temperature; STAI = state-trait anxiety inventory; TSST = trier social stress test.
